# Obesity paradox in stroke – Myth or reality? A systematic review

**DOI:** 10.1371/journal.pone.0171334

**Published:** 2017-03-14

**Authors:** Lisa Oesch, Turgut Tatlisumak, Marcel Arnold, Hakan Sarikaya

**Affiliations:** 1 Stroke Center, Department of Neurology, Bern University Hospital, Bern, Switzerland; 2 Department of Clinical Neuroscience, Institute of Neuroscience and Physiology, Sahlgrenska Academy at University of Gothenburg, Gothenburg, Sweden; McMaster University, CANADA

## Abstract

**Background and purpose:**

Both stroke and obesity show an increasing incidence worldwide. While obesity is an established risk factor for stroke, its influence on outcome in ischemic stroke is less clear. Many studies suggest a better prognosis in obese patients after stroke (“obesity paradox”). This review aims at assessing the clinical outcomes of obese patients after stroke by performing a systematic literature search.

**Methods:**

The reviewers searched MEDLINE from inception to December 2015. Studies were eligible if they included outcome comparisons in stroke patients with allocation to body weight.

**Results:**

Twenty-five studies (299’750 patients) were included and none was randomised. Ten of 12 studies (162’921 patients) reported significantly less mortality rates in stroke patients with higher BMI values. Seven of 9 studies (92’718 patients) ascertained a favorable effect of excess body weight on non-fatal outcomes (good clinical outcome, recurrence of vascular events). Six studies (85’042 patients) indicated contradictory results after intravenous thrombolysis (IVT), however. Several methodological limitations were observed in major part of studies (observational study design, inaccuracy of BMI in reflecting obesity, lacking body weight measurement, selection bias, survival bias).

**Conclusion:**

Most observational data indicate a survival benefit of obese patients after stroke, but a number of methodological concerns exist. No obesity paradox was observed in patients after IVT. There is a need for well-designed randomized controlled trials assessing the effects of weight reduction on stroke risk in obese patients.

## Introduction

Stroke is the leading cause of acquired persistent disability in adults worldwide and the second leading cause of death in patients aged > 60 years. The World Health Organization (WHO) estimates that one new patient suffers stroke each 2 seconds worldwide and one new patient experiences either death or disability every 6 seconds due to stroke. Stroke is responsible for 5.8 million deaths every year, which is more than all deaths due to AIDS, tuberculosis, and malaria together. Therefore, the WHO calls stroke as the incoming epidemic of the 21^st^ century. In addition, recent studies suggest that more and more young patients are hospitalized for stroke—data suggest that about 30% of all patients with stroke are younger than age of 65 years.

Obesity has also reached epidemic dimensions worldwide. The WHO estimates that approximately 2.3 billion adults (31.3%) were overweight and more than 700 million (9.6%) were obese by 2015. Keeping in mind that obesity is an independent predictor of ischemic stroke and especially affects younger patients, obese patients will constitute an increasing group of candidates for stroke treatment and care.

Obesity is an established risk factor for the development of vascular diseases such as stroke. A meta-analysis from 2010 evaluated data from more than 2.2 million participants to address the relationship between excess body weight and stroke incidence.[1] Overweight and obesity were significantly associated with progressively increasing risk of ischemic stroke.[1] It has been shown that each unit increase of BMI was associated with a significant 6% increase in the adjusted relative risk of stroke,[2] the association between BMI and risk of ischemic stroke was linear,[1–5] similar in men and women and regardless of race.[1,3,5,6] However, the impact of excess body weight on prognosis in vascular diseases is controversial. Several studies indicated that overweight and obese patients with heart failure have significantly less mortality rates (both cardiovascular death and all-cause mortality) as compared to the normal-weight counterparts. The in-hospital mortality for patients with decompensated heart failure has been reported to be 10% lower with each 5-unit increase in body-mass-index (BMI).[7] A meta-analysis of 40 studies involving 250’152 patients with coronary artery disease showed that total and cardiovascular mortality was lowest among overweight patients.[8] Meanwhile, many studies suggest better outcomes for obese patients in other diseases or interventions such as chronic heart failure, coronary revascularization, chronic kidney disease, rheumatoid arthritis, chronic obstructive lung disease, or advanced cancers.[9–14] The decreased mortality rate in obese patients is also termed as “obesity paradox” in the literature.[15–17]

While obesity is an established risk factor for stroke, its influence on clinical outcome, mortality, and thrombolysis in acute ischemic stroke is still under debate. Since both obesity and stroke are major public health issues, it is important to fully understand the association between these two diseases. This systematic review aims at assessing the clinical outcome of obese patients after stroke. In addition, the impact of obesity on acute stroke treatment by thrombolysis will be discussed by using a thorough literature search.

## Search strategy and selection criteria

An unlimited PubMed search (MEDLINE) was performed on December 6, 2015, using the MeSH terms: stroke AND obesity OR overweight AND outcome OR mortality, the last two mentioned in title or abstract. This search was combined with two further searches: first using the MeSH term stroke AND obesity paradox, and second using the MeSH terms stroke AND obesity AND thrombolysis, the latter mentioned in title and abstract. In addition, we screened reference lists of all retrieved reports for further publications. Studies were included if they investigated the association of obesity with outcome and mortality after stroke in humans or outcome after intravenous thrombolysis (IVT) with allocation to body weight. Only papers written in English were considered. We excluded studies investigating the association of obesity with outcome and mortality in other diseases than stroke (i.e. cardiovascular diseases, diabetes or sleep apnea). Furthermore, we also excluded studies assessing the association of BMI with outcome after surgical intervention or reports on highly selected subgroups.

Eligibility of all reports was determined by one reviewer (LO) and independently checked by another reviewer (HS) (S1 Fig). Primary outcome measures were functional status expressed by modified Rankin Scale (mRS) and mortality. For obesity measures, we adopted the following BMI threshold categories from WHO: < 18.5 kg/m^2^ for underweight, 18.5 to 24.9 kg/m^2^ for normal weight, 25.0 to 29.9 kg/m^2^ for overweight, and ≥ 30.0 kg/m^2^ for obesity. In many studies investigating the Asian population, BMI thresholds were adapted according to WPRO (WHO Regional Office for the Western Pacific Region) criteria as follows: < 18.5 kg/m^2^ for underweight, 18.5 to 22.9 kg/m^2^ for normal weight, 23.0 to 24.9 kg/m^2^ for overweight, and ≥ 25.0 kg/m^2^ for obesity. We followed the PRISMA guidelines for this systematic review (S1 File).

## Results

We identified 183 unique references through our literature search. After detailed evaluation, 25 studies performed in 299’750 patients fulfilled our eligibility criteria and were included (S1 Fig).

### Obesity and outcome after stroke

We identified 19 studies in 214'708 patients with information on BMI to discuss the association of excess body weight with clinical outcome after ischemic or intracerebral hemorrhage.[18–36] The median number of patients per study was 1791 (range, 365 to 53’812) and the median time of follow-up 30 months (range, 1 week to 11.5 years) (calculated by the mean time of follow-up). Out of these 19 studies, 9 focus on the relationship between BMI and mortality [18–25,27] and 3 on both mortality and non-fatal outcome.[26,30,35] In addition, 6 studies examined further outcomes such as hemorrhagic transformation after acute ischemic stroke, recurrent vascular events, stroke rehabilitation and hospital discharge outcomes.[28,29,31,32,34,36] One study used central obesity as measurement and investigated the prognostic performance of waist-to-height ratio on mortality after acute first-ever stroke.[33]

#### Body mass index and mortality after stroke

Ten of 12 studies evaluating a total of 162’921 patients reported less mortality rates in stroke patients with higher BMI values (S1 Table).[18–22,24–26,30,35] Olsen et al. followed 21’884 patients up to 5 years after stroke (median 1.5 years) and demonstrated first in 2008 that poststroke mortality was inversely associated with BMI.[18] Compared to the cohort with normal body weight (BMI 18.5 to 24.9kg/m^2^), mortality risk was lowest in overweight patients (HR 0.73, 95% CI 0.66–0.81) followed by the cohort of obese patients (HR 0.84, 95% CI 0.74–0.96), whereas the risk of death was increased in under-weight patients (HR 1.63, 95% CI 1.41–1.90).[18] Kim et al. reported comparable findings in 1356 patients with hemorrhagic stroke: long term mortality risk over a mean follow-up duration of 33.6 ± 15.5 months was lowest in overweight and obese patients (HR 0.69, 95% CI 0.49–0.96 and HR 0.61, 95% CI 0.43–0.88, respectively), while underweight patients were at highest risk of death (HR 1.64, 95% CI 1.11–2.40).[21] However, 30-day mortality did not differ between the groups.[21] In a further study, the authors investigated mortality rates in 34’132 Korean patients at 30 days, 90 days, and 1 year after ischemic stroke.[22] Again, an inverse association of BMI with poststroke mortality was observed at 1-year, but not earlier.[22] Skolarus et al. followed 1’791 patients from Texas (US) for about 2 years after acute ischemic stroke and assessed the survival rates.[25] After adjustment for demographics, stroke severity, stroke and mortality risk factors, the relationship between BMI and mortality was U shaped with lowest mortality risk among patients with an approximate BMI of 35 kg/m2, whereas those with lower or higher BMI (<31 kg/m^2^ or >38 kg/m^2^) had higher mortality risk.[25] Zhao et al. investigated 10’905 Chinese patients with ischemic stroke and reported an independent association between mortality and severe obesity at 3 months (OR 2.01, 95% CI 1.12–1.38).[26] In this study, overweight and obese patients had similar mortality rates as compared to the normal-weight reference group, whereas mortality in underweight patients tended to increase again.[26] Doehner et al. studied data from 1521 patients with available BMI who experienced acute stroke or transient ischemic attack (TIA) in Germany.[30] After adjustment for confounding factors, 30-month mortality was highest in underweight patients (HR 2.76, 95% CI 1.75–4.36), decreased continuously in overweight and obese patients (HR 0.86, 95% CI 0.69–1.08 and HR 0.76, 95% CI 0.53–1.10) and was lowest in very obese patients with BMI >35 kg/m^2^ (HR 0.55, 95% CI 0.29–1.02).^21^ In a Danish study by Andersen et al. including 29’326 patients with first-ever acute stroke, proportion of patients who had died within a mean follow up of 2.6 years was lowest in obese patients (HR 0.80; 95% CI 0.73–0.88) and highest in underweight patients (HR 1.66; 95% CI 1.49–1.84) as compared to normal-weight ones.[35] These associations were significant after adjustment for age, gender, civil status, stroke severity, and risk factors.[35] Vemmos et al. recruited 2785 patients with first-ever stroke in Greece.[20] Based on BMI estimation, obese and overweight stroke patients had significantly better early and long-term survival rates compared to those with normal BMI although NIHSS score on admission was similar among the groups.[20] Overweight and obese patients had a significantly lower risk of 10-year mortality compared to normal-weight patients after adjusting for all confounding variables (HR 0.82; 95% CI, 0.71–0.94 and HR, 0.71; 95% CI, 0.59–0.86, respectively).[20]

Towfighi et al followed up 644 stroke survivors from an U.S. survey and noted stroke survivors to be more likely overweight or obese.[19] Furthermore, they found an age-dependent effect of obesity on post-stroke mortality: the association of higher BMI with mortality risk was strongest in younger individuals and declined linearly with increasing age, such that in the elderly aged > 70 years, excess of body weight had a rather protective effect.[19] The authors concluded that younger stroke patients would especially benefit from vigorous efforts to prevent obesity.[19] Bell et al. reported similar results with lower poststroke mortality in overweight and obese older women aged 50 to 79 years.[24] In contrary, women with BMI <18.5 kg/m^2^ before stroke had double the mortality risk of normal weight women, even after controlling for smoking, cancer, diabetes, cardiovascular risk factors, and physical activity.[24]

Ryu et al. followed up 1’592 consecutive Asian patients with ischemic stroke for a median of 4 years.[23] The level of BMI was inversely related to initial neurological severity in linear regression analysis (p = 0.002).[23] After adjustment of all covariates including initial neurologic severity and age, an independent association between BMI and poststroke mortality was found in underweight (HR: 2.79, 95% CI 1.92–4.05), but not in overweight or obese patients.[23]

The research group of Olsen and colleagues, who first reported obesity paradox in stroke patients,[18] investigated in a subsequent study the relationship between BMI and death by the index stroke within the first week or month.[27] While BMI was inversely related to mean age at stroke onset, no difference in the risk for death by stroke was observed in the first week or first month among overweight and obese patients as compared to the reference group with normal weight.[27] Again, underweight patients were at increased risk of death.[27]

To summarize the main results, risk of mortality in obese vs. normal weight patients from abovementioned studies are separately illustrated in S1 Fig (note that follow-up time significantly varied among studies).

#### Waist-to-height-ratio (WHtR) and mortality after stroke

Chiquete et al. compared WHtR and BMI as adiposity measures by tetrapolar bioimpedance analysis and found WHtR to better correlate with total fat mass.[33] The authors thereafter analyzed WHtR as predictor of mortality in 821 Mexican patients with ischemic stroke and reported a U-shaped relationship between baseline WHtR and mortality. On multivariate analysis, baseline WHtR ≤ 0.300 or >0.800 independently predicted 12-month all-cause mortality, whereas BMI was not associated with mortality.[33]

#### Body mass index and non-fatal outcome after stroke

Nine studies evaluated non-fatal outcome in 92’718 stroke patients (mean number of patients per study 10’302, range 365 to 29’326).[26,28–32,34–36] Seven of these 9 studies ascertained a favorable effect of excess body weight on non-fatal outcomes such as recurrence of stroke, myocardial infarction, or vascular death, favorable outcome defined as modified Rankin Scale (mRS) score 0–1, need for institutional care, or functional progress in stroke rehabilitation (S2 Table).[26,29,31,32,34,35,37]

Kim et al. investigated 365 Korean patients with acute ischemic stroke and showed a decrease in the occurrence of hemorrhagic transformation with increasing BMI.[31] As compared to normal weight, obesity was independently associated with lower risk of hemorrhagic transformation after acute ischemic stroke (OR: 0.39, 95% CI 0.17–0.87).[31] However, the proportion of cardioembolic strokes and use of thrombolysis was higher in patients with hemorrhagic transformation, whereas BMI showed an inverse correlation with stroke severity measured by NIHSS score.[31]

In TEMPiS trial, the risk of stroke recurrence at 30 months after the index event (ischemic or hemorrhagic stroke, TIA) in obese patients tended to be lower than in those with underweight or normal weight, but the differences did not reach statistical significance (3.7%, 9.7% and 7.9%, respectively; p = 0.178).[30] The trial also investigated non-fatal and functional outcomes which were worst in underweight patients and improved by increasing BMI.[30] Thus, obese patients had a lower risk of institutional care and dependency than patients with normal weight or underweight. [30] On the contrary, underweight patients had consistently the highest risks for all endpoints (death, recurrent stroke, need for institutional care and functional impairment).[30]

Post hoc analysis from the Prevention Regimen for Effectively Avoiding Second Strokes (PRoFESS) Trial, which was a randomized controlled multicenter trial including 20’246 patients, showed comparable risks for stroke recurrence at 2.5 years in lean, overweight and obese patients after adjusting for confounders.[29] However, overweight and obesity were significantly associated with lower risk of major vascular events (recurrent stroke, myocardial infarction, or vascular death) as compared with the lean group (HR: 0.84%, 95% CI 0.77–0.92 and HR: 0.86%, 95% CI 0.77–0.96, respectively).[29] Andersen et al. investigated the association between BMI and risk of stroke recurrence by evaluating 28’382 patients’ data retrospectively and showed that overweight and obese patients had less frequently a history of previous stroke as compared to normal-weight patients (OR: 0.89, 95% CI 0.83–0.96 and OR: 0.90, 95% CI 0.82–0.98).[34] In a prospective Danish survey reported by the same authors, the risk of readmission for recurrent stroke within a median follow-up time of 2.6 years was significantly lower in obese patients as compared to normal-weight patients (HR: 0.84, 95% CI 0.72–0.92).[35]

Similar to findings in TEMPiS trial, Zhao et al. reported better 3-month functional recovery in overweight and obese stroke patients when compared with normal-weight counterparts (OR: 1.24, 95% CI 1.12–1.38 and OR: 1.15, 95% CI 0.99–1.34, respectively).[26] In line with these findings, Burke et al. observed that functional progress in stroke rehabilitation was fastest in overweight patients.[32] Kim et al. investigated 2679 Korean patients with ischemic stroke and also stated better 3-month functional outcomes 3 months in overweight and obese patients.[36] However, the association lost its significance after adjusting for stroke severity measured by NIHSS score at admission.[36]

In opposite to these studies indicating a favorable impact of higher BMI levels on clinical outcome, Razinia et al. reported that obese patients may have lesser chances for being discharged home after stroke and tended to stay longer in hospital.[28] However, BMI at admission had no influence on functional activity at hospital discharge.[28]

### Obesity and outcome after thrombolysis in ischemic stroke

We identified 6 studies on 85’042 patients (range 169–81’579) reporting on the outcome of obese stroke patients treated with IVT (S3 Table).[38–43] The examined clinical endpoints were functional outcome, which was defined as favorable (mRS score 0 or 1), good (mRS 0–2) or poor (mRS 3–6). In addition, the following endpoints were measured: symptomatic and asymptomatic intracranial hemorrhages, discharge destination, and mortality. We could not identify studies assessing the relationship between obesity and stroke outcome after intra-arterial thrombolysis or mechanical thrombectomy.

Sarikaya et al. were the first to assess the outcome of obese stroke patients after treatment with IVT.[41] As compared to non-obese patients (n = 251) with BMI < 30 kg/m^2^, the obese counterparts (n = 53) had significantly higher rates of mortality (13.2% vs. 4.2%) and reached markedly less often a favorable outcome (50.9% vs. 68.1%) at 3 months, whereas the rates of symptomatic and asymptomatic intracranial hemorrhages were comparable in both groups.[41] Furthermore, multivariate analyses identified obesity as an independent predictor of unfavorable clinical outcome and mortality after IVT.[41] In a subsequent study, the same working group compared the outcomes of patients weighing > 100 kg (n = 95) with those weighing ≤ 100 kg (n = 1384).[40] No significant differences were observed in the 2 groups regarding the endpoints favorable outcome, good outcome, mortality, or symptomatic intracranial hemorrhage (sICH).[40] After multivariable adjustments, however, body weight > 100 kg was independently associated with mortality in patients treated with IVT.[40] Diedler et al. assessed data of 27’910 patients registered in Safe Implementation of Treatment in Stroke–International Stroke Thrombolysis Register (SITS-ISTR).[38] After adjustment for baseline characteristics, the odds ratio for favorable outcome was similar in patients ≤100 kg and >100 kg, but patients >100 kg (n = 1’190) had again a significantly higher risk for mortality (OR: 1.37, 95% CI 1.08–1.74).[38] Seet et al. observed in their study on 169 patients that frequency of poor functional recovery and sICH were very similar among obese, overweight, and normal weight patients after IVT.[42] The authors assumed that the number of metabolic risk components may contribute more significantly to functional recovery.[42] A national US survey assessed outcome data of 81’579 stroke patients treated with IVT.[39] The analyses were adjusted for age and gender, but not for clinical stroke severity.[39] Obese patients (n = 5174) had similar rates for favorable outcome and lower odds of intracerebral hemorrhage and in-hospital mortality as compared to non-obese, but they were also more likely to be discharged with moderate to severe disability.[39] In the study by Seo et al. assessing 321 Korean patients treated with IVT, age and NIHSS score were significantly higher in underweight patients.[43] Thus, being underweight was independently associated with poorer long-term survival as compared to patients with normal body weight.[43]

## Discussion

We identified a total of 25 studies looking for an association between excess body weight and clinical outcome after stroke in 299’750 patients. The vast majority of studies reported a decreased mortality rate in overweight or obese patients, while a favorable effect of excess body weight (BMI > 25kg/m^2^) on poststroke outcome was attributed. Of note, these associations mostly remained statistically significant after adjusting for confounding risk factors. These results hint to an obesity paradox in stroke in terms of higher survival rates and better functional outcome in stroke patients with excess body weight. No obesity paradox was observed for acute stroke patients treated with IVT, whereas no data were available for intra-arterial thrombolysis or mechanical thrombectomy.

The association between obesity and favorable outcome after stroke was strong and consistent in many studies. The findings are in analogy to observations in other diseases such as coronary artery disease and chronic heart failure.[8,9] However, these data need to be cautiously interpreted. First, all studies were observational and no randomized controlled trial was identified, thus the causal relationship cannot be judged.[44] In similarity, a Cochrane review from 2009 identified no randomized trial to establish the impact of weight reduction on stroke incidence.[45] Furthermore, there is neither a biologically graded nor a linear relationship between the degree of obesity and risk of mortality. Thus, mortality was lowest rather in overweight patients and significantly higher in obese and underweight counterparts in many studies.[18,25,26,33] Likewise, overweight and not obese patients had the best functional recovery in stroke rehabilitation.[32] Therefore, the results do not allow the conclusion “the fatter, the better”. Another explanation for “obesity paradox” in stroke may be that time of follow-up in cited studies (range 1 week to 11.5 years, median 30 months) was too short to detect detrimental effects of obesity. In comparison, harmful impact of obesity on cardiovascular outcomes in Framingham-Study was evident after a follow-up period of 8 years in men and 14 years in women.[46] Moreover, underweight patients have a higher prevalence of co-morbid conditions such as chronic infections or malignant tumors explaining the poor prognosis and higher mortality (reverse causation).[10,47] It is assumed that the higher mortality in underweight patients is rather caused by severe medical co-morbidities whereas mortality in obese patients is primarily explained by cardiovascular events. Another point of view is the catabolic post-stroke metabolism leading to additional weight loss.[48] This might not only be caused by impaired feeding, inactivity, and paralysis resulting in sarcopenia, but also co-regulated by other factors and pathways that may be abnormally activated or impaired after stroke. So, several stroke-associated mechanisms such as stress-related neuroendocrine autonomic nervous activation, pro-inflammatory cytokines, increased oxygen-free radical load, and systemic hormonal imbalances may promote an overall catabolic state.[48] Thus, obese patients with better metabolic reserve may be less affected from this unfavorable metabolic dysregulation as compared to underweight patients. [15,37]

Furthermore, certain confounders could have statistically distorted the included study results. Ischemic stroke has different etiologies depending on body weight and age.[49] In our study, obese patients were of younger age, while infarct volumes were often small due to higher proportions of lacunar infarcts. As a consequence, stroke severity was rather low in these patients. On the contrary, patients with lower or normal BMI values tended to be older and stroke was often caused by atrial fibrillation, which is associated with larger infarct volumes, high stroke severity, and thus increased mortality risk. [22,23,25,35,49] Of note, younger age and lower stroke severity are the most crucial prognostic factors for favorable outcome after stroke. Ryu et al. criticized that many studies reporting obesity paradox did not adjust for stroke severity.[23] They observed an inverse relationship between BMI and long-term mortality in multivariate analyses, but also an inverse association between BMI and stroke severity. Therefore, the initial stroke severity was assumed to be a mediator between levels of BMI and poststroke mortality, contradicting the existence of obesity paradox. [23] Similar findings were also reported by Kim and colleagues: the initially observed association between excess body weight and good outcome was no more significant after adjustment for stroke severity.[36] Additional data from literature indicate that obese patients are frequently younger at the time of first cardiovascular event, leading to milder symptoms at earlier presentation and allowing prevention measurements in an early stage. Treatment bias may be another cause of obesity paradox as it has been shown that physicians treat obese patients more aggressively than lean patients due to assumed increase of vascular risk. Thus, obese patients were more often treated with antithrombotics, antihypertensive drugs, and statins though comparable risk profile.[50]

A further methodological limitation concerns the measurement of obesity. Although BMI was the most commonly used tool for this purpose, its diagnostic accuracy is highly questionable.[51] As compared to body fat percent (BF%) estimated by bioelectrical impedance analysis, BMI ≥ 30 kg/m^2^ had a high specificity of 96%, but a poor sensitivity of 43% in assessment of obesity. Consequently, half of the obese participants were missed for identification. Moreover, body mass index generally correlates well with BF %, but is unable to differ between BF% and lean mass. Additionally, the diagnostic performance of BMI diminishes by increasing age.[51] Recent data suggest that alternative tools such as waist-to-hip ratio or waist circumference to be more precise in the measurement of obesity. Of note, obesity paradox was no more existent when BMI was replaced by waist-to-hip ratio.[52]

The obesity paradox could also be a result of statistical distortion due to a survival bias. Olsen et al. first reported an obesity paradox in stroke patients, but questioned their observation in a second survey later on and discussed whether the findings resulted from a selection bias.[18]^,^ [27] If obese stroke patients suffered from stroke or any disease of less severity than normal-weight stroke patients, patients in higher BMI levels would have a lower mortality risk in any disease and so falsely imply a survival advantage of excess body weight. Thus, when estimating the independent effect of BMI on poststroke mortality risk by multivariate statistics, stroke severity and comorbid variants have to be adjusted for. However, this is only possible when death was caused by the index stroke. Differences between groups in mortality risk due to various diseases cannot reliably be adjusted for by multivariate analyses, mainly because the severity of these diseases had not been quantified (e.g. by using the Charlson comorbidity index).[53] Dehlendorff et al. hypothesized, that death by stroke was only caused by the index stroke if it occurred within first week or first month after the stroke.[27] Thus when studying deaths occurring within the selected time period, risk related to death by index stroke did not differ significantly in overweight, obese, and normal-weight patients.[27] Furthermore, publication bias may be another reason for obesity paradox. The majority of studies were retrospective and therefore differences in data quality may also distort the results. This could partially explain the findings in IVT (lack of obesity paradox), where data are collected prospectively and data quality is suspected to be higher than in retrospective studies. In addition, observational studies may be hampered by inclusion bias as patients dying early after stroke were probably excluded due to lack of body weight measurement.

From a biological point of view, obesity paradox could be explained by potentially protective effects of adipose tissue, which is now increasingly acknowledged as a major endocrine organ.[9] Adipose tissue secretes soluble TNF-α receptors and may so neutralize the biologic impact of TNF-α. Moreover, obese individuals have increased levels of serum lipid levels which could bind and detoxify endotoxin-lipoproteins and consequently block the release of inflammatory cytokines.[9] Both mechanisms may impede the poststroke pro-inflammatory state.[9]

Data on obesity and its impact on clinical outcome after IVT are rather limited and incongruent. Whereas some studies suggested higher mortality rates in obese patients, others did not find a significant difference depending on body weight of patients. This discrepancy needs to be clarified by further prospective large-scale studies as obese patients might be prone to more in-hospital complications such as venous thromboembolism and the clot-dissolving effect of alteplase may be hampered by plasminogen activator inhibitor-1 which seems to be overexpressed in adipose tissue.[54,55] In general, no obesity paradox was observed for patients treated with IVT. Moreover, the risk for sICH and probability for favorable outcome were comparable within different body weight groups. These data support the current weight-adapted dose regimen of alteplase with upper dose limit. A main drawback of all studies was that body weight and height were rather estimated by relatives of patients or their caregivers than measured. It has been shown, that estimation of body weight before thrombolysis may result in dosing errors in one-third of patients.[56] In addition, some studies had rather low sample size or did not adjust for clinical stroke severity, which is a main determinant of mortality and clinical prognosis.[39,41,42] Further studies should also focus at clinical outcome in underweight patients, which is also an increasing group by older age with assumed high risk for medical complications and mortality (e.g. through malnutrition or overdosing of drugs). Finally, our literature search was limited to PubMed and did not consider other databases such as EMBASE or Cochrane Library.

## Conclusion

In summary, most observational data indicate a survival benefit of obese patients after stroke, but a number of methodological concerns exist. Obesity is a well-proven independent risk factor for occurrence of stroke, and weight reduction in overweight or obese patients is still recommended for primary stroke prevention. We would also stick to the same recommendation in younger patients after occurrence of stroke (secondary prevention) with respect to longer life expectancy and detrimental cardiovascular effects of obesity over years. No obesity paradox was observed in patients after IVT, thus there is no need to change the current dosage scheme of alteplase in obese patients. On the other hand, there is a need for well-designed and adequately-powered randomized controlled trials assessing the effects of weight reduction on stroke occurrence and recurrence in obese patients. Furthermore, future observational studies should be population-based, prospective and account for methodological limitations of the former studies (inaccuracy of BMI in measuring obesity, false estimation of body weight, lacking adjustment for comorbidities).

## Supporting information

S1 Fig Study Flow Diagram. (PDF)

S1 File Supplemental PRISMA Guidelines. (DOCX)

S1 Table Studies addressing Body Mass Index and mortality after stroke. (PDF)

S2 Table Studies addressing Body Mass Index and non-fatal outcome after stroke. (PDF)

S3 Table Studies addressing Obesity and outcome after thrombolysis in ischemic stroke. (PDF)
