# Supplementary figures and images for: Obesity paradox in stroke – Myth or reality? A systematic review
Source: PLoS One. 2017 Mar 14;12(3):e0171334. doi: 10.1371/journal.pone.0171334 (PMC5349441; doi:10.1371/journal.pone.0171334)

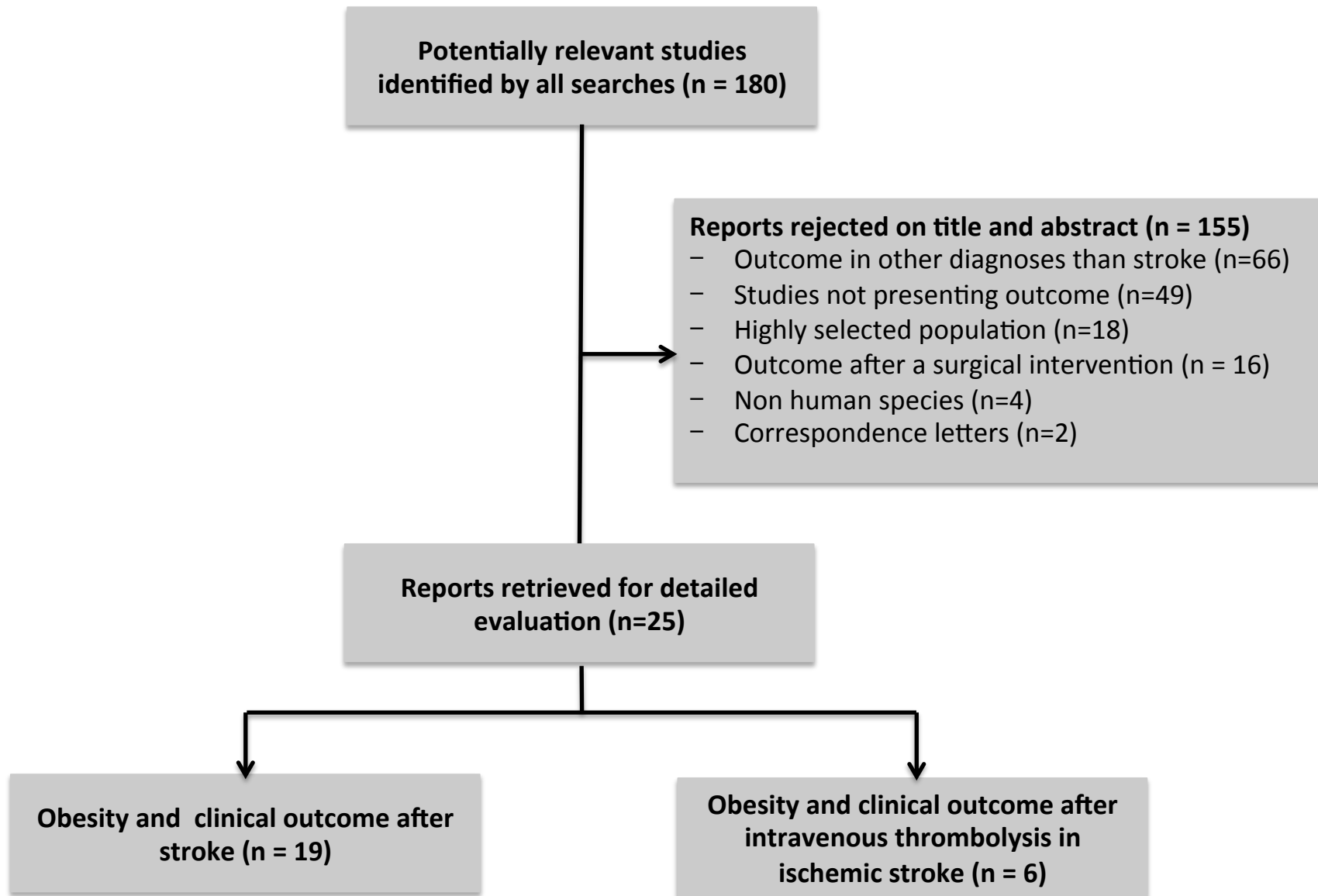

Supplement: S1 Fig — (PDF) [file pone.0171334.s001.pdf]
