# Supplementary material for: Obesity paradox in stroke – Myth or reality? A systematic review
Source: PLoS One. 2017 Mar 14;12(3):e0171334. doi: 10.1371/journal.pone.0171334 (PMC5349441; doi:10.1371/journal.pone.0171334)
Supplement: S1 File — (DOCX) [file pone.0171334.s002.docx]

**CHECKLIST PRISMA GUIDELINES**

**TITLE**

**Title**reported on page 1

**ABSTRACT**

**Structured summary**
reported on page 2

**INTRODUCTION**

**Rationale**

reported on page 3, lines 2 ff.

**Objectives**

reported on page 4, lines 1 ff.

**METHODS**

**Protocol and registration**

There is no registration for this systematic review.

**Eligibility criteria**

reported on page 5, lines 1 ff.

**Information sources**

reported on page 5, lines 1 ff.

**Search**

reported on page 5, lines 1 ff. and Supplemental Material.

**Study selection**

reported on page 5, lines 7 ff.

**Data collection process**

reported on page 5, lines 9 ff.

**Data items**

reported on page 5, lines 10 ff.

**Risk of bias in individual studies**

discussed on page 13, lines 33 ff.

**Summary measures**

not applicable (systematic review, no meta-analysis)

**Synthesis of results**

not applicable (systematic review, no meta-analysis)

**Risk of bias across studies**

reported on page 13, lines 33 ff.

**Additional analyses**

not performed

**RESULTS**

**Study selection**

reported on page 6, lines 2 ff. and in Supplemental Material (Study Flow Diagram)

**Study characteristics**

Supplemental Material: reported in Table 1, Table 2 and Table 3

**Risk of bias within studies**

not applicable (systematic review, no meta-analysis)

**Results of individual studies**

Supplemental Material: reported in Table 1, Table 2 and Table 3

**Synthesis of results**

not applicable (systematic review, no meta-analysis)

**Risk of bias across studies**

not applicable (systematic review, no meta-analysis)

**Additional analysis**

none

**DISCUSSION**

**Summary of evidence**

reported on page 12, lines 2 ff.

**Limitations**

reported on page 12, lines 13 ff.

**Conclusions**

reported on page 15, lines 3 ff.

**FUNDING**

**Funding**

reported on page 1
