# Supplementary material for: Obesity paradox in stroke – Myth or reality? A systematic review
Source: PLoS One. 2017 Mar 14;12(3):e0171334. doi: 10.1371/journal.pone.0171334 (PMC5349441; doi:10.1371/journal.pone.0171334)
Supplement: S1 Table — (PDF) [file pone.0171334.s003.pdf]

S1 Table. Studies addressing Body Mass Index and mortality after stroke

**Study**	**Patients with information on Body Mass Index (n)**	**Follow-Up**	**Risk of Mortality**		**Factors adjusted for in multivariate analysis**
Olsen, 2008[1] (Denmark)	21'884 patients with acute stroke	5 year mortality (Median Follow-up 1.5 years)	Underweight: HR: 1.63 (95% CI 1.41 – 1.90)		Age, Sex, Civil Status, stroke severity (Scandinavian Stroke Scale score), cardiovascular risk factors
			Normal-weight: HR: 1 (reference)		
			Overweight: HR: 0.73 (95% CI 0.66-0.81)		
			Obese: HR: 0.84 (95% CI 0.74 – 0.96)		
			Severely Obese: HR: 0.84 (95% CI 0.64 – 1.10)		
Towfighi, 2009[2] (USA)	644 patients with baseline history of stroke	All - cause mortality rate ratios per kg/m^2 relative to person of same age	Age 40: HR: 1.05 (95% CI 0.99 – 1.11)		Age, Hypertension, Diabetes, Hypercholesterolemia, Smoking, History of myocardial infarction
		(Median Follow up 11.5 years)	Age 80: HR: 0.95 (95% CI 0.95 – 0.98)		
Vemmos, 2011[3] (Greece)	2785 patients with first-ever acute stroke	Cumulative probability of Survival (%) (Median Follow-Up 47 months)	1 wk	Normal-weight: 90.2 (95% CI 88.4 – 92.0)	-
				Overweight: 92.8 (95% CI 91.2 – 94.4)	
				Obese: 96.4 (95% CI 94.8 – 97.9)	
			10 yr	Normal-weight: 41.5 (95% CI 39.7 – 45.0)	
				Overweight: 47.4 (95% CI 43.5 – 51.3)	
				Obese: 10 yr: 52.5 (95% CI 46.4 – 58.6)	
		10 yr mortality	Normal-weight: HR: 1 (reference)		Age, Discharge Treatment

			Overweight: HR: 0.84 (95% CI 0.73 - 0.97)	
			Obese: HR: 0.74 (95% CI 0.61 – 0.90)	
Kim, 2011[4] (South Korea)	1356 patients with hemorrhagic stroke	30 – day mortality	Underweight: HR: 0.75 (95% CI 0.26 – 2.11)	Age at study entry, diabetes, Glasgow Coma Scale Score, surgical management, systolic blood pressure, glucose, volume of hematoma, extension of hemorrhage into ventricles
			Normal-weight: HR: 1 (reference)	
			Overweight: HR: 0.86 (95% CI 0.47 – 1.56)	
			Obese: HR: 0.89 (95% CI 0.47 – 1.68)	
		Long - term mortality (Mean Follow-Up 33.6 months)	Underweight: HR: 1.64 (95% CI 1.11 – 2.40)	Age at study entry, diabetes, Glasgow Coma Scale Score, glucose, volume of hematoma and extension of hemorrhage into ventricles
			Normal-weight: HR: 1 (reference)	
			Overweight: HR: 0.69 (95% CI 0.49 – 0.96)	
			Obese: HR: 0.61 (95% CI 0.43 – 0.88)	
Ryu, 2011[5] (South Korea)	1592 patients with ischemic stroke	Long - term all-cause mortality (Median Follow-Up 4 years)	Underweight: HR: 2.79 (95% CI 1.92 – 4.05)	Age, sex, previous stroke, hypertension, diabetes, current smoking, heart disease, NIHSS score categorized into 0-1, 2-3, 4-6, and ≥7
			Normal-weight: HR: 1 (reference)	
			Overweight: HR: 0.95 (95% CI 0.73 – 1.25)	
			Obese: HR: 0.76 (95% CI 0.57 – 1.01)	
Kim, 2012[6] (South Korea)	34132 patients with acute ischemic stroke	Mortality rate ratios per kg/m^2 increase of BMI (Mean Follow-Up 32.6 months)	HR: 0.96 (95% CI 0.95 – 0.97)	-

		Mortality at various time points after onset of ischemic stroke	30 d	BMI < 18.5: HR: 1.04 (95% CI 0.85 – 1.27)	-
				BMI 20 – 23: HR: 1 (reference)	
				BMI 23 – 25: HR: 1.05 (95% CI 0.90 – 1-22)	
				BMI 27.5 – 30: HR: 1.23 (95% CI 0.95 – 1.59)	
			90 d	BMI < 18.5: HR: 1.21 (95% CI 1.06 – 1.39	
				BMI 20 – 23: HR: 1 (reference)	
				BMI 23 – 25: HR: 0.98 (95% CI 0.87 – 1.11)	
				BMI 27.5 – 30: HR: 1.06 (0.86 – 1.30)	
			1 yr	BMI < 18.5: HR: 1.30 (95% CI 1.17 – 1.44)	
				BMI 20 – 23: HR: 1 (reference)	
				BMI 23 – 25: HR: 0.90 (95% CI 0.83 – 0.99)	
				BMI 27.5 – 30: HR 0.92 (0.79 – 1.07)	
Doehner, 2013[7] (Germany)	1521 patients with TIA or stroke	Death at 30 months Follow -Up	Underweight: HR: 2.76 (95% CI 1.75 – 4.36)		Age, sex, living in partnership prior to the event, co-morbidities, stroke severity, classification of the cerebral event (TIA vs. ischemic stroke vs. intracerebral hemorrhage), assignment to the Telestroke Unit or conventional treatment arm of the TEMPiS study protocol
			Normal weight: HR: 1.0 (reference)		
			Overweight: HR: 0.86 (95% CI 0.69 – 1.08)		
			Obese: HR: 0.76 (95% CI 0.53 – 1.10)		
			Very obese: HR: 0.55 (95% CI 0.29 – 1.02)		
Bell, 2013[8]	3173	Mortality (Follow-Up time	Underweight: HR: 2.02 (95% CI 0.98 – 4.16)		Age, marriage, social support

(USA)	postmenopausal women aged 50 – 79 with stroke	unknown)	Normal weight: HR: 1.0 (reference)<br>Overweight: HR: 0.72 (95% CI 0.58 – 0.90)<br>Obese: HR: 0.69 (95% CI 0.53 – 0.88)	score, income, diabetes, hypertension, smoking status, pre-stroke physical activity, pre-stroke functional ability, incident stroke type, prevalent conditions pre-stroke, post-stroke Glasgow outcome scale
Skolarus, 2014[9] (USA)	1791 Patients with acute ischemic stroke ≥ 45 years	All-cause mortality in acute ischemic stroke (Median Follow-Up 660 days)	-<br>-	Age, race/ ethnicity, sex, stroke severity, hypertension, atrial fibrillation, coronary artery disease, diabetes mellitus, heart failure, high cholesterol chronic obstructive pulmonary disease, dementia, end-stage renal disease, cancer, history of stroke or transient ischemic attack, excessive alcohol use, smoking
Zhao, 2014[10] (China)	10905 patients with t acute ischemic stroke	All-cause mortality at 3-month Follow-Up	Underweight: OR: 1.30 (95% CI 0.93 – 1.82)<br>Normal-weight: OR: 1 (reference)<br>Overweight: OR: 1.06 (95% CI 0.89 – 1.28)<br>Obese: OR: 1.06 (95% CI 0.81 – 1.38)<br>Severely obese: OR 2.01 (95% CI 1.10 – 3.69)	Age, gender, stroke severity (NIHSS), prestrike mRS, AIS Trial of ORG 10172 in Acute Stroke Treatment subtype, and risk factors or comorbidities (including hypertension, diabetes,

					dyslipidemia, coronary heart disease, atrial fibrillation or flutter, heart failure, current or previous smoking, and history of stroke)
Dehlendorff, 2014[11] (Denmark)	53’812 patients with acute stroke	Mortality at 1 week and 1 month Follow-Up	1 wk	Underweight: HR: 1.41 (95% CI 1.13 – 1.76)	Age, sex, stroke type, stroke severity (Scandinavian Stroke Scale score), cardiovascular risk factors, civil status, socioeconomic status
				Normal weight: HR: 1.0 (reference)	
				Overweight: HR: 0.85 (95% CI 0.69 – 1.04)	
				Obese: HR: 1.19 (95% CI 0.92 – 1.52)	
			1 m	Underweight: HR: 1.55 (95% CI 1.37 – 1.76)	
				Normal weight: HR: 1.0 (reference)	
				Overweight: HR: 0.88 (95% CI 0.78 – 0.98)	
				Obese: HR: 0.98 (95% CI 0.84 – 1.15)	
Andersen, 2015[12] (Denmark)	29’326 patients with acute first-ever stroke	Mortality (Median Follow-Up 2.6 years)	Underweight: HR: 1.66 (95% CI 1.49 – 1.84)		Cardiovascular risk factors, age, gender, civil status, stroke severity (Scandinavian Stroke Scale score),
			Normal weight: HR: 1.0 (reference)		
			Overweight: HR: 0.72 (95% CI 0.68 – 0.78)		
			Obese: HR: 0.80 (95% CI 0.73 – 0.88)		

HR: Hazard Ratio, OR: Odds Ratio, RR: Relative Risk, CI: Confidence Interval, - : no data available in study, wk: week, m: month, yr: year

1. Olsen TS, Dehlendorff C, Petersen HG, Andersen KK (2008) Body mass index and poststroke mortality. Neuroepidemiology 30: 93-100.
2. Towfighi A, Ovbiagele B (2009) The impact of body mass index on mortality after stroke. Stroke 40: 2704-2708.
3. Vemmos K, Ntaios G, Spengos K, Savvari P, Vemmou A, et al. (2011) Association between obesity and mortality after acute first-ever stroke: the obesity-stroke paradox. Stroke 42: 30-36.

4. Kim BJ, Lee SH, Ryu WS, Kim CK, Lee J, et al. (2011) Paradoxical longevity in obese patients with intracerebral hemorrhage. Neurology 76: 567-573.
5. Ryu WS, Lee SH, Kim CK, Kim BJ, Yoon BW (2011) Body mass index, initial neurological severity and long-term mortality in ischemic stroke. Cerebrovasc Dis 32: 170-176.
6. Kim BJ, Lee SH, Jung KH, Yu KH, Lee BC, et al. (2012) Dynamics of obesity paradox after stroke, related to time from onset, age, and causes of death. Neurology 79: 856-863.
7. Doehner W, Schenkel J, Anker SD, Springer J, Audebert HJ (2013) Overweight and obesity are associated with improved survival, functional outcome, and stroke recurrence after acute stroke or transient ischaemic attack: observations from the TEMPiS trial. Eur Heart J 34: 268-277.
8. Bell CL, LaCroix A, Masaki K, Hade EM, Manini T, et al. (2013) Prestroke factors associated with poststroke mortality and recovery in older women in the Women's Health Initiative. J Am Geriatr Soc 61: 1324-1330.
9. Skolarus LE, Sanchez BN, Levine DA, Baek J, Kerber KA, et al. (2014) Association of body mass index and mortality after acute ischemic stroke. Circ Cardiovasc Qual Outcomes 7: 64-69.
10. Zhao L, Du W, Zhao X, Liu L, Wang C, et al. (2014) Favorable functional recovery in overweight ischemic stroke survivors: findings from the China National Stroke Registry. J Stroke Cerebrovasc Dis 23: e201-206.
11. Dehlendorff C, Andersen KK, Olsen TS (2014) Body mass index and death by stroke: no obesity paradox. JAMA Neurol 71: 978-984.
12. Andersen KK, Olsen TS (2015) The obesity paradox in stroke: lower mortality and lower risk of readmission for recurrent stroke in obese stroke patients. Int J Stroke 10: 99-104.
