# Supplementary material for: Obesity paradox in stroke – Myth or reality? A systematic review
Source: PLoS One. 2017 Mar 14;12(3):e0171334. doi: 10.1371/journal.pone.0171334 (PMC5349441; doi:10.1371/journal.pone.0171334)
Supplement: S2 Table — (PDF) [file pone.0171334.s004.pdf]

S2 Table. Studies addressing Body Mass Index and non-fatal outcome after stroke.

Study	Patients with information on Body Mass Index (n)	Outcome	Results	Factors adjusted for in multivariate analysis
Razinia, 2007[1] (USA)	451 Patients with ischemic stroke aged > 18 or older	Favorable discharge destination, home vs. other (Follow-up unknown)	Normal-weight: 1 (reference)	Age, sex, history of hypertension, atrial fibrillation, diabetes mellitus, coronary artery disease, statin use, stroke severity (NIHSS), systolic blood pressure, cardioembolic stroke mechanisms, current smoking
			Overweight: OR: 1.13 (95% CI 0.68 – 1.91)	
			Obese: OR: 0.46 (95% CI 0.22 – 0.96)	
			Severely Obese: OR: 0.42 (95% CI 0.13 – 1.37)	
		Length of hospital stay, Median hospital stay in days (Follow-up unknown)	Normal-weight: 5.2 (95% CI 4.6 – 5.8)	
			Overweight: 5.2 (95% CI 4.6 – 5.8)	
			Obese: 6.4 (95 % CI 5.4 – 7.5)	
			Severely Obese: 6.3 (95% CI 4.5 – 8.2)	
Ovbiagele, 2011[2] (USA, UK, Germany)	20246 patients aged 55 years, or those aged 50 to 54 years with at least 2 additional vascular risk factors, who experienced an ischemic stroke < 120 days before	Recurrent stroke (Follow-Up 2.5 years)	Normal-weight: 1 (reference)	Age, sex, previous stroke, diabetes, myocardial infarction, baseline systolic blood pressure, hypertension, qualifying stroke – small vessel disease, qualifying stroke – cardioembolic, current smoking status, stroke severity (NIHSS),
			Overweight: HR: 0.947 (95% CI 0.850 -1.056)	
			Obese: HR: 0.947 (95% CI 0.8333 – 1.077)	

	randomization and whose condition was stable			previous transient ischemic attack
		Major vascular event (Stroke, myocardial infarction, vascular death) (Follow-Up 2.5 years)	Normal-weight: 1 (reference)	Age, sex, previous stroke, diabetes, myocardial infarction, baseline systolic blood pressure, hypertension, qualifying stroke – small vessel disease, qualifying stroke – cardioembolic, hyperlipidemia, coronary artery disease current smoking status, antihypertensive medication at baseline, stroke severity (NIHSS), previous transient ischemic attack, Asian ethnicity
			Overweight: HR: 0.841 (95% CI 0.769 – 0.918)	
			Obese: HR: 0.860 (95% CI 0.774 – 0.956)	
Andersen, 2013[3] Denmark	28'382 patients with acute stroke	Risk of stroke being recurrent (Follow-Up unknown)	Underweight: OR: 1.23 (95% CI 1.06 – 1.42)	Age, sex, stroke severity (Scandinavian Stroke Scale), stroke type, civil status, cardiovascular risk factor
			Normal weight: OR: 1 (reference)	
			Overweight: OR: 0.89 (95% CI 0.83 – 0.96)	
			Obese: OR: 0.90 (95% CI 0.82 – 0.98)	
Doehner, 2013[4]	1521 patients with acute stroke or TIA	Recurrent stroke or death at 30 months after stroke	Underweight: OR: 2.74 (95% CI 1.23 – 6.03)	Age, sex, living in partnership prior to the event,
			Normal weight: OR: 1.0 (reference)	

(Germany)			Overweight: OR: 0.79 (95% CI 0.60 – 1.03)	co-morbidities, stroke severity, classification of the cerebral event (TIA vs. ischemic stroke vs. intracerebral hemorrhage), assignment to the Telestroke Unit or conventional treatment arm of the TEMPiS study protocol
			Obese: OR: 0.56 (95% CI 0.37 – 0.86)	
			Very obese: OR 0.51 (95% CI 0.27 – 0.97)	
		Functional disability (high dependency)	Underweight: OR: 1.28 (95% CI 0.50 – 3.25)	
			Normal-weight: OR: 1.0 (reference)	
			Overweight: OR: 0.74 (95% CI 0.50 – 1.00)	
			Obese: OR: 0.60 (95% CI 0.39 – 0.91)	
			Very obese: OR: 0.68 (95% CI 0.37 – 1.25)	
		Institutional care	Underweight: OR: 2.18 (95% CI 0.90 – 5.28)	
			Normal-weight: OR: 1.0 (reference)	
			Overweight: OR 0.68 (95% CI 0.51 – 0.91)	
			Obese: OR 0.60 (95% CI 0.38 – 0.92)	
			Very obese: OR 0.49 (95% CI 0.25 – 0.99)	
Kim, 2013[5] (South Korea)	365 patients with first-ever acute ischemic stroke	Hemorrhagic transformation within 1 week after acute ischemic stroke with/ without thrombolytic treatment	Underweight: OR: 1.05 (95% CI 0.28 – 3.96)	Age, gender, hypertension, diabetes, hyperlipidemia, current smoking, initial NIHSS score, thrombolysis, acute heparin treatment, stroke subtype, previous aspirin use, previous warfarin use, presence of advanced WMLS and cerebral micro bleeds
			Normal-weight: OR: 1.0 (reference)	
			Overweight: OR: 0.48 (95% CI 0.21 – 1.11)	
			Obese: OR: 0.39 (95% CI 0.17 – 0.87)	

Zhao, 2014[6] (China)	10905 patients with acute ischemic stroke	Favorable 3 – month functional recovery	Underweight: OR: 1.24 (95% CI 0.96 – 1.59)<br>Normal-weight: 1 (reference)<br>Overweight: OR: 1.24 (95% CI 1.12 – 1.38)<br>Obese: OR: 1.15 (95% CI 0.99 – 1.34)<br>Severely obese: OR: 1.07 (95% CI 0.72 – 1.60)	Age, gender, stroke severity (NIHSS), prestrike mRS, AIS Trial of ORG 10172 in Acute Stroke Treatment subtype, and risk factors or comorbidities (including hypertension, diabetes, dyslipidemia, coronary heart disease, atrial fibrillation or flutter, heart failure, current or previous smoking, and history of stroke)
Burke, 2014[7] (USA)	819 patients admitted to an acute freestanding rehabilitation hospital with a diagnosis of stroke	Functional progress in stroke rehabilitation (mean FMI efficiency)(unknown Follow-up)	Underweight: 1.01 (95% CI 0.6 – 1.43)<br>Normal-weight: 1.29 (95% CI 1.15 – 1.49)<br>Overweight: 1.38 (95% CI 1.22 – 1.55)<br>Obese: 1.05 (95% CI 0.82 – 1.23)	Age, sex, length of hospital stay
Andersen, 2015[8] (Denmark)	29'326 patients with acute first-ever stroke	Risk of readmission for recurrent stroke (Median Follow-up 2.6 years)	Underweight: HR 1.04 (95% CI 0.81 – 1.34)<br>Normal weight: HR 1.0 (reference)<br>Overweight: HR: 0.97 (95% CI 0.87 – 1.08)<br>Obese: HR: 0.84 (95% CI 0.72 – 0.97)	Cardiovascular risk factors, age, gender, civil status, stroke severity (Scandinavian Stroke Scale)
Kim,	703 patients with	3-month functional outcome	BMI ≤ 21.2: OR: 1.0 (reference)	Gender, age, dyslipidemia,

2015[9] (South Korea)	ischemic stroke	(modified Rankin Scale)	BMI = 21.2 – 23.0: OR: 0.57 (95% CI 0.30 – 1.07)	smoking, atrial fibrillation, serum white blood cell, serum hematocrit, serum fasting blood sugar, diastolic blood pressure, stroke subtype, initial neurological severity (NIHSS)
			BMI = 23.1 – 24.5: OR 0.60 (95% CI 032 – 1.14)	
			BMI= 24.6 – 26.2: OR: 0.43 (95% CI 0.21 – 0.86)	
			BMI ≥ 26.3: OR: 0.76 (95% CI 0.39 – 1.49)	

HR: Hazard Ratio, OR: Odds Ratio, RR: Relative Risk, CI: Confidence Interval, - : no data available in study, wk: week, m: month, yr: year

1. Razinia T, Saver JL, Liebeskind DS, Ali LK, Buck B, et al. (2007) Body mass index and hospital discharge outcomes after ischemic stroke. Arch Neurol 64: 388-391.
2. Ovbiagele B, Bath PM, Cotton D, Vinisko R, Diener HC (2011) Obesity and recurrent vascular risk after a recent ischemic stroke. Stroke 42: 3397-3402.
3. Andersen KK, Olsen TS (2013) Body mass index and stroke: overweight and obesity less often associated with stroke recurrence. J Stroke Cerebrovasc Dis 22: e576-581.
4. Doehner W, Schenkel J, Anker SD, Springer J, Audebert HJ (2013) Overweight and obesity are associated with improved survival, functional outcome, and stroke recurrence after acute stroke or transient ischaemic attack: observations from the TEMPiS trial. Eur Heart J 34: 268-277.
5. Kim CK, Ryu WS, Kim BJ, Lee SH (2013) Paradoxical effect of obesity on hemorrhagic transformation after acute ischemic stroke. BMC Neurol 13: 123.
6. Zhao L, Du W, Zhao X, Liu L, Wang C, et al. (2014) Favorable functional recovery in overweight ischemic stroke survivors: findings from the China National Stroke Registry. J Stroke Cerebrovasc Dis 23: e201-206.
7. Burke DT, Al-Adawi S, Bell RB, Easley K, Chen S, et al. (2014) Effect of body mass index on stroke rehabilitation. Arch Phys Med Rehabil 95: 1055-1059.
8. Andersen KK, Olsen TS (2015) The obesity paradox in stroke: lower mortality and lower risk of readmission for recurrent stroke in obese stroke patients. Int J Stroke 10: 99-104.
9. Kim Y, Kim CK, Jung S, Yoon BW, Lee SH (2015) Obesity-stroke paradox and initial neurological severity. J Neurol Neurosurg Psychiatry 86: 743-747.
