# Supplementary material for: Obesity paradox in stroke – Myth or reality? A systematic review
Source: PLoS One. 2017 Mar 14;12(3):e0171334. doi: 10.1371/journal.pone.0171334 (PMC5349441; doi:10.1371/journal.pone.0171334)
Supplement: S3 Table — (PDF) [file pone.0171334.s005.pdf]

S3 Table. Studies addressing Obesity and outcome after thrombolysis in ischemic stroke

Study	Patients (n)	Outcome	Results		Factors adjusted for in multivariate analysis
Diedler, 2011[1] (Germany, Sweden, Italy, Slovak Republic)	27'910 patients registered in Safe Implementation of Treatment in Stroke–International Stroke Thrombolysis Register (SITS-ISTR), 1190 (4.3%) weighed >100kg	Major neurological improvement	2h	OR: 0.92 (95% CI 0.76 – 1.12)	Age, sex, history of hypertension, diabetes mellitus, hyperlipidemia, smoking, stroke subtypes (lacunar vs other), baseline National Institutes of Health Stroke Scale score (NIHSS), baseline blood glucose, blood pressure, alteplase dose (mg/kg)
			24h	OR: 1.12 (95% CI 0.97 – 1.20)	
		Incidence of symptomatic intracerebral hemorrhage	Per SITS MOST	OR: 1.60 (95% CI 1.06 – 2.41)	
			Per ECASS II	OR: 1.08 (95% CI 0.81 – 1.43)	
		Functional outcome at 3 months	mRS 0 - 1	OR: 1.01 (95% CI 0.87 – 1.18)	
			mRS 0 - 2	OR: 0.99 (95% CI 0.84 – 1.17)	
		Mortality at 3 months	OR: 1.32 (95% CI 1.08 – 1.74)		
Sarikaya, 2011[2] (Switzerland)	304 patients with acute ischemic stroke who underwent IVT with alteplase but no other or additional	Death at 3 months	Non-obese: 4.0 %		Age, gender, hypertension, diabetes mellitus, smoking status, baseline National Institutes of Health Stroke Scale score (NIHSS), time from
			Obese: 13.2 %		
		Favorable outcome at 3 months	Non-obese: 68.1 %		
			Obese: 50.9 %		
		Symptomatic intracranial hemorrhage at 3 months	Non-obese: 1.6 %		
			Obese: 1.9 %		

	thrombolytic treatment				stroke onset to treatment, prestroke treatment with antiplatelet agents
Sarikaya, 2011[3] (Switzerland)	1479 patients with acute ischemic stroke who underwent IVT	Favorable outcome at 3 – months (mRS score 0 or 1)	Weight > 100kg: 45.3%		Baseline National Institutes of Health Stroke Scale score (NIHSS), age, gender, blood glucose, diastolic blood pressure, antiplatelet medication, systolic blood pressure
			Weight < 100kg: 47.6%		
			OR: 0.715 (95% CI 0.422 – 1.213)		
		Good outcome at 3 – months (mRS score 0 – 2)	Weight > 100kg: 57.9%		
			Weight < 100kg: 63.9%		
			OR: 0.776 (95% CI 0.265 – 0.807)		
		Mortality rate at 3 months	Weight > 100 kg: 16.8%		
			Weight < 100 kg: 12.3%		
			OR: 1.445 (95% CI 1.305 – 5.341)		
		Rate of symptomatic intracranial hemorrhage (SICH)	NINDS definition	Weight > 100kg: 1.1%	
				Weight < 100kg: 5.4%	
				OR 0.190 (95% CI 0.041 – 2.251)	
			ECAS II Definition	Weight > 100kg: 1.1%	
				Weight < 100kg: 4.6%	
				OR: 0.372 (95% CI 0.049 – 2.790)	
Hassan, 2013[4] (USA)	Total of 81579 patients admitted with acute ischemic stroke	No to minimal disability	OR: 1.0 (95% CI 0.8 – 1.1)		Age, sex, diabetes, hypertension renal failure, hospital location/ teaching
		Moderate to severe disability	OR: 1.2 (95% CI 1.0 – 1.3)		
		Mortality	OR: 0.6 (95% CI 0.5 – 0.8)		
		ICH secondary to	OR: 0.7 (95% CI 0.5 – 0.9)		

	undergoing thrombolysis, of these 5174 (6.3%) patients categorized as obese	thrombolytic treatment		status, APR-DRG severity scale
Seo, 2013[5] (South Korea)	321 patients treated with thrombolysis therapy, including intravenous recombinant tissue plasminogen activator and/or intra-arterial thrombolysis	Long-term mortality	Underweight: HR: 3.49 (95% CI 1.44 – 8.46)	Age, sex, hypertension, atrial fibrillation, baseline National Institutes of Health Stroke Scale score on admission (NIHSS), thrombolysis methods, current smoking status, homocysteine, low-density lipoprotein, apopolipoprotein B, free T4
			Normal weight: Reference	
			Overweight: HR: 0.66 (95% CI 0.33 - 1.34)	
			Obese: HR: 1.35 (95% CI 0.66 – 2.78)	
Seet, 2014[6] (USA, Singapore)	169 patients with acute ischemic stroke who received intravenous	Symptomatic intracerebral hemorrhage	Normal-weight: OR: 1 (reference)	Age, stroke severity (Baseline National Institutes of Health Stroke Scale score (NIHSS)), stroke
			Overweight: OR: 0.94 (95% CI 0.52 – 5.12)	
			Obese: OR: 0.82 (95% CI 0.63 – 4.94)	
		Poor functional recovery	Normal-weight: OR: 1 (reference)	
			Overweight: OR: 1.12 (95% CI 0.54 – 6.21)	

	thrombolysis within 3 hours of symptom onset		Obese: OR: 1.08 (95% CI 0.45 - 4.21)	subtype

HR: Hazard Ratio, OR: Odds Ratio, RR: Relative Risk, CI: Confidence Interval, - : no data available in study, wk: week, m: month, yr: year

1. Diedler J, Ahmed N, Glahn J, Grond M, Lorenzano S, et al. (2011) Is the maximum dose of 90 mg alteplase sufficient for patients with ischemic stroke weighing >100 kg? Stroke 42: 1615-1620.
2. Sarikaya H, Elmas F, Arnold M, Georgiadis D, Baumgartner RW (2011) Impact of obesity on stroke outcome after intravenous thrombolysis. Stroke 42: 2330-2332.
3. Sarikaya H, Arnold M, Engelter ST, Lyrer PA, Mattle HP, et al. (2011) Outcome of intravenous thrombolysis in stroke patients weighing over 100 kg. Cerebrovasc Dis 32: 201-206.
4. Hassan AE, Chaudhry SA, Jani V, Grigoryan M, Khan AA, et al. (2013) Is there a decreased risk of intracerebral hemorrhage and mortality in obese patients treated with intravenous thrombolysis in acute ischemic stroke? J Stroke Cerebrovasc Dis 22: 545-549.
5. Seo JH, Jeong HY, Noh S, Kim E-G, Ji K-H, et al. (2013) Relationship of Body Mass Index and Mortality for Acute Ischemic Stroke Patients after Thrombolysis Therapy. J Neurocrit Care 6: 92-96.
6. Seet RC, Zhang Y, Wijdicks EF, Rabinstein AA (2014) Thrombolysis outcomes among obese and overweight stroke patients: an age- and National Institutes of Health Stroke Scale-matched comparison. J Stroke Cerebrovasc Dis 23: 1-6.
